# How Shall We Manage the Pedicle in Supra-Vaginal Hysterectomy?

**Published:** 1887-11

**Authors:** 


### Editorial.

### *HOW SHALL WE MANAGE THE PEDICLE IN SUPRA-VAGINAL HYSTERECTOMY?*

While the question of the treatment of the pedicle in ovariotomy may be said to be already decided in favor of the intra-peritoneal method, not so with reference to its management in supra-vaginal hysterectomy, which is still under judgment. The two conditions differ so widely, both anatomically and pathologically, that it is difficult to understand how they could ever have been regarded as identical, or even analogous, as to the principles which should govern their management.

So much of success depends upon the technique of the operation in abdominal sections of the class which has for its object the excision of the pelvic organs of women, that it must necessarily be discussed in detail—point by point, item by item, step by step. At one time, it is the ligature, either as to its substance or the method of its application, which receives attention; at another, drainage, both as to its method and whether it shall be employed at all; and, again, the cautery becomes the theme of discussion—and so on unto the end of the long catalogue. It is only in this way that truth can be evolved, and so we reach the solution of great problems by this steady-going, siege-like process.

The chief sources of danger from the pedicle in the operation in question are: First, hemorrhage, and second, sepsis; and these are especially difficult to overcome, if the pedicle be short and thick. In these respects, the stump widely differs from the ovarian pedicle.

To prevent hemorrhage during the operation, Martin, of Berlin, throws an india-rubber tube around the pedicle, claiming that no blood need be lost, excepting that which is in the tumor itself; he then unites the stump, by excising the tissue in such a manner as to leave it cone-shaped, and drops the pedicle. This may be said to approach nearer to the ideal than the extra-peritoneal method of dealing with the pedicle, for it leaves the parts nearer restored to their normal relations.

Dr. George Granville Bantock has recently called attention anew to this subject in a paper read at the late meeting of the American Gynecological Society, detailing the improvements he has made in the management of the pedicle through a series of fifty-seven supra-vaginal hysterectomies treated by the extra- and five by the intra-peritoneal method. Of the former, forty-five recovered and twelve died; of the latter, one recovered and four died. This is a rare experience, and enables a man who can boast it to speak from the chair. He began his operations by severing the pedicle with the cautery according to Baker Brown, but the patient died from hemorrhage, and he never tried this plan again. He now employs Koeberlé's serre-nœud as modified by himself, using soft annealed iron wire to constrict the pedicle, which is tightened with the clock-key ratchet. If the wire is properly adjusted at first, it will rarely be necessary to tighten it again for four or five days, but should there be oozing at any time, a turn or two of the key will control it. He has even allowed two weeks to elapse before disturbing the dressings, especially if the stump remain dry, but always changes them whenever they become moist.

In one of his successful cases, he removed the pregnant uterus together with a fibro-cyst weighing thirteen pounds. In another case, his forty-fifth, he applied the serre-nœud just below the level of the ovaries, partially tightening it, then included the broad ligament in long hemostatic forceps, thus securing the large vessels. The peritoneal covering of the uterus and tumor was then divided three inches from the wire loop and reflected towards the latter. Tightening the loop, the pins were inserted an inch from the wire, and a second constrictor was applied just behind the pins, after which the first was removed. This illustrates his method of gaining length in cases where the pedicle is short, and thus relieving the strain on the broad ligaments. He regards the treatment of the stump in supra-vaginal hysterectomy as a very different thing from that of the ovarian pedicle—a point which seems to be well taken.

Mr. Thornton's method of operating, including the treatment of the pedicle, does not differ materially from Dr. Bantock's, excepting in the employment of antiseptics. As is well known, the latter dispenses with Listerism, while the former carries it out fully, including the spray. Mr. Thornton also applies styptic iron powder to the stump, which Dr. Bantock thinks unnecessary.

The intra-peritoneal method may be said to be the favorite of the German school led by Martin and others, while the extra-peritoneal method is the usual one followed by the English gynecological surgeons. In America, most operators incline to the extra-peritoneal plan, believing that it is safer in our present light, and economizes time withal, an element of importance many times. This practice is likely to prevail pending the bringing forward of the yet-to-be-discovered ideal method, which Dr. Bantock very frankly admits has not, up to the present time, been accomplished.

